# A survival nomogram involving nutritional-inflammatory indicators for cervical cancer patients receiving adjuvant radiotherapy

**DOI:** 10.7150/jca.100564

**Published:** 2024-09-09

**Authors:** Shanshan Wang, Mengli Zhao, Zhongrong Gao, Xiaojing Yang, Yudong Wang, Keqin Hua, Jie Fu

**Affiliations:** 1 Department of Radiation Oncology, Shanghai Sixth People's Hospital Affiliated to Shanghai Jiao Tong University School of Medicine, Shanghai, China.; 2 Department of Gynecologic Oncology the International Peace Maternity and Child Health Hospital School of Medicine, Shanghai Jiao Tong University, Shanghai 200025, China.; 3 Department of Gynecology, Obstetrics and Gynecology Hospital of Fudan University, 419 FangXie Road, Shanghai 200011, China.

**Keywords:** nutrition, inflammation, prognostic model, cervical cancer, postoperative radiotherapy

## Abstract

**Objective:** The combined impact of nutritional and inflammatory status on survival of cervical cancer patients remained unclear. This study aimed to construct a survival nomogram involving both nutritional and inflammatory indicators and evaluate their potential correlation.

**Methods:** This retrospective study included 325 cervical cancer patients who received adjuvant radiotherapy between September 2010 and September 2020. Baseline nutritional indicators such as body mass index (BMI), controlling nutritional status (CONUT) and serum albumin were assessed. Inflammatory indicators of platelet/lymphocyte ratio (PLR), neutrophil/lymphocyte ratio (NLR), systemic immune inflammation index (SII) and system inflammation response index (SIRI) were evaluated respectively. The LASSO regression and Cox regression models were applied for variable selection and nomogram building. The predictive accuracy and superiority of prognostic model were assessed by area under curve (AUC), C-index, decision curve analysis (DCA), integrated discrimination improvement (IDI) and net reclassification improvement (NRI).

**Results:** Patients with high inflammatory indicators (PLR, NLR and SII) and poor nutritional status (CONUT scores > 2) suffered poorer prognosis compared to these with well nutritional status and lower inflammation levels. Our study unveiled a positive correlation between malnutrition and hyperinflammation. Even after accounting for baseline inflammatory level, malnutrition remained a significant risk factor for patients. Notably, the inflammatory level and nutritional status were further modulated by the clinical features of patients. Patients with poorer nutritional status exhibited higher levels of PLR, NLR, SII and SIRI, particularly for those in advanced clinical stages and with non-squamous cell carcinoma. In addition, our study found elevated levels of circulating basophil and serum carbohydrate antigen 125 (CA125) were associated with the poor prognosis. The prognostic nomogram which incorporated the nutritional-inflammatory indicators of PLR and CONUT showed a favorable performance with the AUC value of 0.76 at 5-year survival prediction. The DCA, IDI and NRI consistently demonstrated the favorable superiority of the model. Moreover, the nomogram-based risk stratification system could effectively classify patients into three mortality risks subgroups.

**Conclusions:** Poorer nutritional and high inflammatory status collectively contributed to the poorer prognosis. The prognostic nomogram which incorporated nutritional-inflammatory indicators significantly improved the prediction of long-term outcomes of cervical cancer patients undergoing adjuvant radiotherapy.

## Introduction

Cervical cancer (CC) continues to be a significant global health burden, ranking fourth in both cancer incidence and mortality among women^1^. Postoperative adjuvant radiotherapy had significantly improved the overall survival (OS) of CC patients with adverse pathological factors after surgery^2^. However, approximately 10-20% of these patients still suffered poor outcomes, with a 5-years OS around 75%^3^. The International Federation of Gynecology and Obstetrics (FIGO) staging system was commonly utilized to provide guidance for the choice of treatment and evaluation of prognosis^4^. However, observed disparities in prognosis within similar stages highlighted the limitations of relying solely on FIGO staging system^4^, ^5^. Identifying high-risk patients and implementing individualized treatment strategies to improve the prognosis remained a challenge for clinicians.

Malnutrition was associated with the poor clinical outcomes of many malignant tumors including CC^6^-^10^. Traditionally, laboratory nutritional biomarkers were widely used for the assessment of malnutritional risk, such as serum albumin, retinol-binding protein (RBP) and serum cholesterol^11^. Building upon these serum nutritional biomarkers, multifactorial nutritional assessment tools like the Controlling Nutritional Status (CONUT) and Prognostic Nutritional Index (PNI) had been developed, offering simple and effective assessment methods for hospitalized patients. Prior research indicated that high-risk of malnutrition assessed by CONUT and PNI were associated with poor prognosis in CC patients receiving postoperative radiotherapy^12^, ^13^. Nutrition and inflammation were intricately intertwined, jointly influencing the clinical outcomes of cancer patients^14^. Tumor-associated inflammatory response served as a pivotal driving force behind malnutrition of patients^15^. Alterations in peripheral circulating blood cells count and distribution mirrored the systemic inflammatory response, typically manifested by neutrophilia, thrombocytosis, and relative lymphopenia^16^. Previous studies have highlighted the potential of inflammatory indicators evaluated by complete blood count (CBC) as prognostic factors in cervical cancer^5^. The prognostic value of platelet/lymphocyte ratio (PLR) had gained significant attention in recent researches with widespread applications in various cancers including CC^17^, ^18^. Previous researches indicated that patients with high inflammatory levels of systemic immune inflammation index (SII) or system inflammation response index (SIRI) experienced worse clinical outcomes in CC^19^, ^20^. Moreover, other multiparametric inflammatory indicators, such as the lymphocyte-to-monocyte ratio (LMR) and neutrophil-to-lymphocyte ratio (NLR), had been confirmed as promising prognostic indicators in CC^21^, ^22^.

Despite accumulating studies had highlighted the prognostic value of the inflammatory and nutritional indicators in CC. However, the majority of published risk analysis reports were primarily limited to identifying "which is a risk factor". This study aimed to explore the correlation between nutritional and inflammatory indicators and investigate their combined impact on CC patients receiving postoperative radiotherapy.

## Materials and methods

### Study population

This retrospective study ultimately enrolled 325 CC patients who received postoperative adjuvant radiotherapy from September 2010 to September 2020. Radiotherapy was performed approximately 4 weeks after radical hysterectomy. Pathological staging was reassigned according to the 2018 FIGO guidelines. All patients were treated with external beam pelvic irradiation delivered by using intensity-modulated radiation therapy (IMRT). The clinical target volume encompassed the upper vagina, paracervical tissues, and the common, external, and internal iliac, presacral and obturator lymph node regions, as defined by the Radiation Therapy Oncology Group guidelines for whole pelvis radiotherapy. The radiation dose was delivered in fractions of 1.8-2.0 Gy, for a total average dose of 50.4 Gy. Concurrent chemotherapy utilized a platinum-based regimen. Adjuvant brachytherapy was administered postoperatively to patients with positive or close surgical margins.

Patients who met the inclusion criteria were eventually enrolled in the study cohort. Inclusion and exclusion criteria: (1) Patients with cervical cancer who have undergone postoperative adjuvant radiotherapy were included into the study population. (2) Patients with the absence of baseline data were excluded from the study population. (3) Patients who had incompletely or inaccurately documented medical history were excluded. (4) Patients with severe endocrine and metabolic diseases, as well as infectious conditions were excluded from the study population. (5) Patients with autoimmune, or severe hepatic or renal disease were excluded from the study population. (6) Cases of lost to follow-up were excluded. A follow-up schedule was implemented, with visits occurring every three months for the first two years post-treatment, followed by annual visits, including hospital and local healthcare facility reviews, outpatient visits, inpatient assessments, and telephonic consultations. This study aimed to assess OS as the primary clinical outcome which was defined as the time from initiation of radiotherapy to death or the end of follow-up.

### Data collection

In this study, data extracted for each patient involved 42 potential prognostic variables (detailed in Table S1). The selected clinical and morphologic characteristics included age at onset, histological subtypes, and FIGO stages. Due to the release of the new version of the staging, we adjusted the staging based on the actual situation of the patient according to the 2018 version of the FIGO staging system. We further categorized the FIGO stages into the following three groups, including IB2-IB3, IIA1/IIA2/IIB and IIIC1/IIIC2.

The current study included the following nutritional-inflammatory indicators (detailed in Table S2): total protein, albumin, prealbumin, serum creatinine, retinol binding protein, total cholesterol, BMI, CONUT scores, prognostic nutritional index (PNI), platelet-to-lymphocyte (PLR), neutrophil-to-lymphocyte (NLR), monocyte -to- lymphocyte (MLR), systemic immune inflammation index (SII), system inflammation response index (SIRI), white blood cell count, red blood cell count, hemoglobin concentration, platelet count, lymphocyte count, monocyte count, neutrophil count, basophil count, eosinophil count, lymphocyte percentage, monocyte percentage, neutrophil percentage, basophil percentage, eosinophil percentage, mean corpuscular volume (MCV), mean corpuscular hemoglobin (MCH), mean platelet volume (MPV), platelet distribution width (PDW), red cell distribution width (RDW), platelet hematocrit (PCT). The CONUT score was a nutritional screening tool that incorporated serum albumin, total cholesterol, and lymphocyte count. It ranged from 0 to 12, with higher scores indicating worse nutritional status: 0-1 = normal, 2-4 = mild malnutrition, 5-8 = moderate malnutrition, and 9-12 = severe malnutrition^23^. Table S3 provided detailed definitions of multiparameter nutritional-inflammatory indicators such as SII and PNI.

The serum tumor markers incorporated in the current study included carcinoembryonic antigen (CEA, ng/mL), carbohydrate antigen 125 (CA125, U/mL), carbohydrate antigen 153 (CA153, U/mL), carbohydrate antigen 199 (CA199, U/mL) and squamous cell carcinoma antigen (SCC-Ag, ug/mL).

ALL of the abovementioned parameters were test and evaluated in all patients with 1 week before radiotherapy. In order to optimize sensitivity and specificity and improve the overall accuracy and utility of the test, the optimal cutoff values of continuous variables were determined by the maximum Youden index (sensitivity + specificity - 1) to convert them into high and low group (detailed in Table S1).

### Statistical analysis

Statistical analyses were performed using R (version 4.3.2) and GraphPad Prism (version 10.1.2). Youden index (sensitivity + specificity - 1) was used to identify the optimal cut-off points for continuous variables like age and lymphocyte count. Baseline characteristics were compared between different cohorts using Chi-square and Fisher's exact tests. Survival analysis was performed by using the univariate Cox regression and Kaplan-Meier method. The Log Rank Test was used in survival analysis to compare the survival distributions of groups. The LASSO Cox regression and bidirectional stepwise multivariate Cox regression analysis were employed to construct the prognostic nomogram model. Schoenfeld residuals was employed to assess the validity of the Proportional Hazards Assumption. Multicollinearity was evaluated using variance inflation factors (VIFs; cut-off > 2). The AUC, C-index, and calibration curves were employed to assess the predictive accuracy of the prognostic model. The nomogram's potential for clinical benefit and its incremental value in risk prediction were assessed by using DCA, NRI and IDI. Student's t-test and Mann-Whitney U test were employed to analyze differences between groups. A significant difference was considered if the two-sided *P*-value was < 0.05.

## Results

### Clinical characteristics of study population

With a median follow-up of 50 months, the overall survival rates of study patients were 86.9%, 83.6%, and 76.4% at 3, 4, and 5 years, respectively (Figure S1). The primary clinical characteristics of the entire study cohort were summarized in Table 1. The median age of patients were 50 years at diagnosis, ranging from 26 to 76 years. Squamous cell carcinoma was the most prevalent histological subtype (83.4%). Adenocarcinoma and other histological types were observed in 29 and 25 cases, respectively. Patients were regrouped according to the FIGO 2018 guideline, with stage IIIC1 being the most frequent (31.7%). Lymph node metastasis was observed in 114 patients. A comprehensive overview of clinical features of total cohort were provided in Table S1. The enrolled patients were randomly divided into the training group (251 cases) and the validation group (124 cases) at a ratio of 2:1. No significant differences in baseline nutritional-inflammatory indicators were observed between the training and testing cohorts. (Table S2).

### Survival difference between patients with different nutritional and inflammatory status

**Figure 1:**
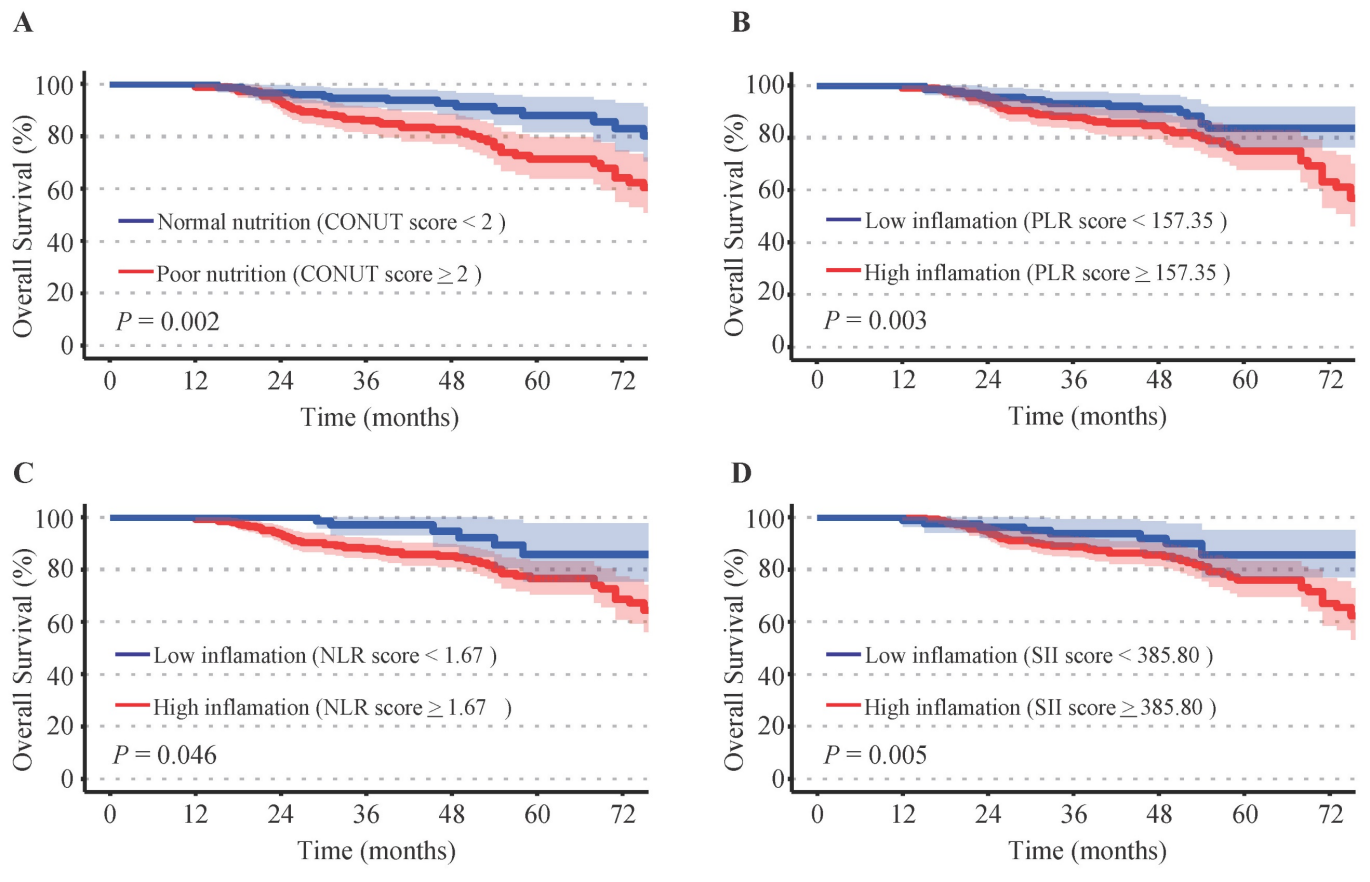
Kaplan-Meier survival analysis. (**A**) Controlling Nutritional Status (CONUT) score, (**B**) platelet-to-lymphocyte ratio (PLR), (**C**) neutrophil-to-lymphocyte ratio (NLR), (**D**) systemic immune inflammation index (SII).

Elevated inflammatory indicators of PLR (*P* = 0.003), NLR (*P* = 0.046), and SII (*P* = 0.005) were associated with shorter overall survival rates. Patients with mild or moderate malnutrition (CONUT scores ≥ 2) suffered poorer prognosis compared to those with normal nutritional status (CONUT scores < 2) (*P* = 0.002). Kaplan-Meier survival curves illustrated the survival differences between patients with different levels of nutrition and inflammation (Figure 1).

### Construction of the prognostic model and risk stratification system

**Figure 2:**
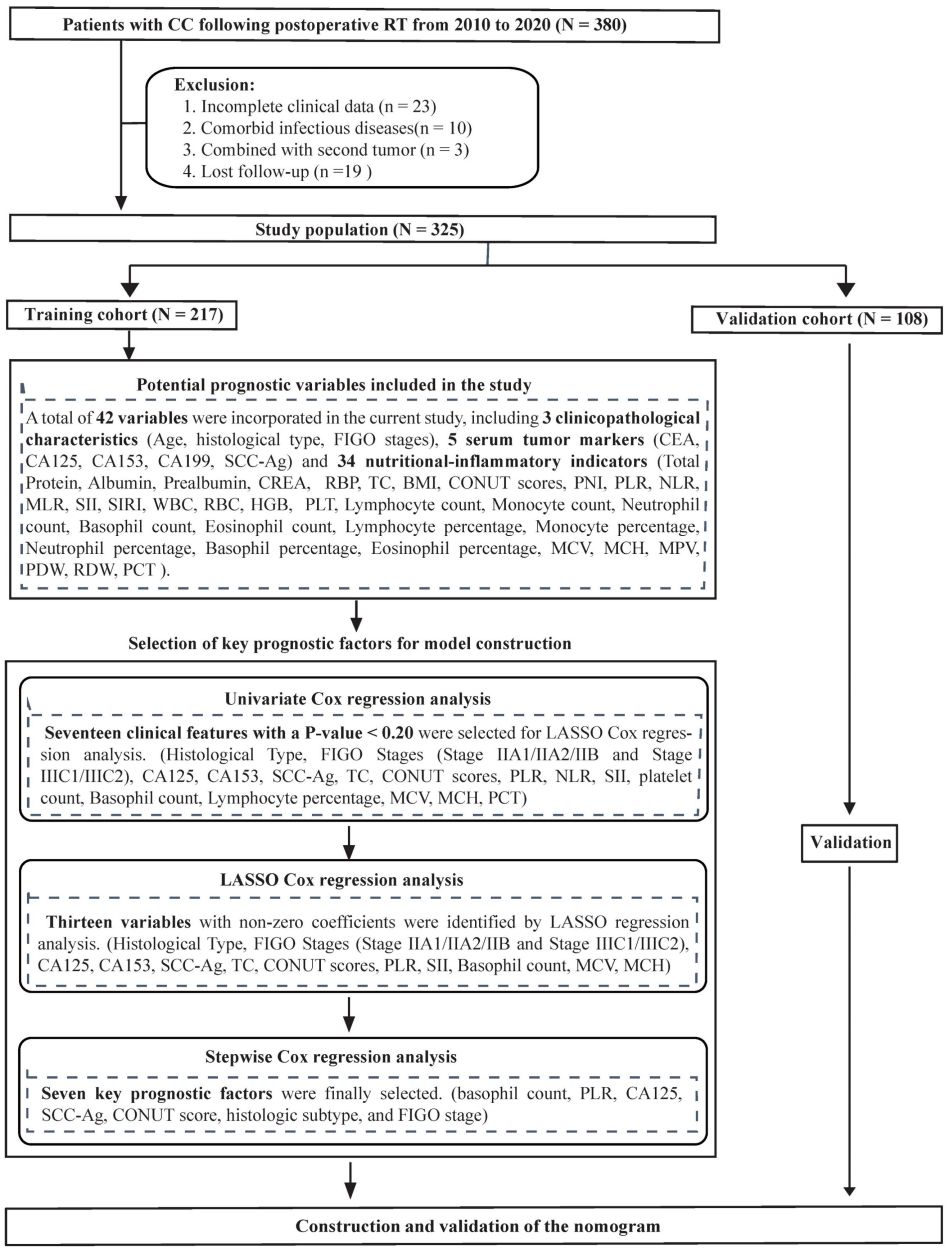
Study flow of constructing the prognostic nomogram model.

**Figure 3:**
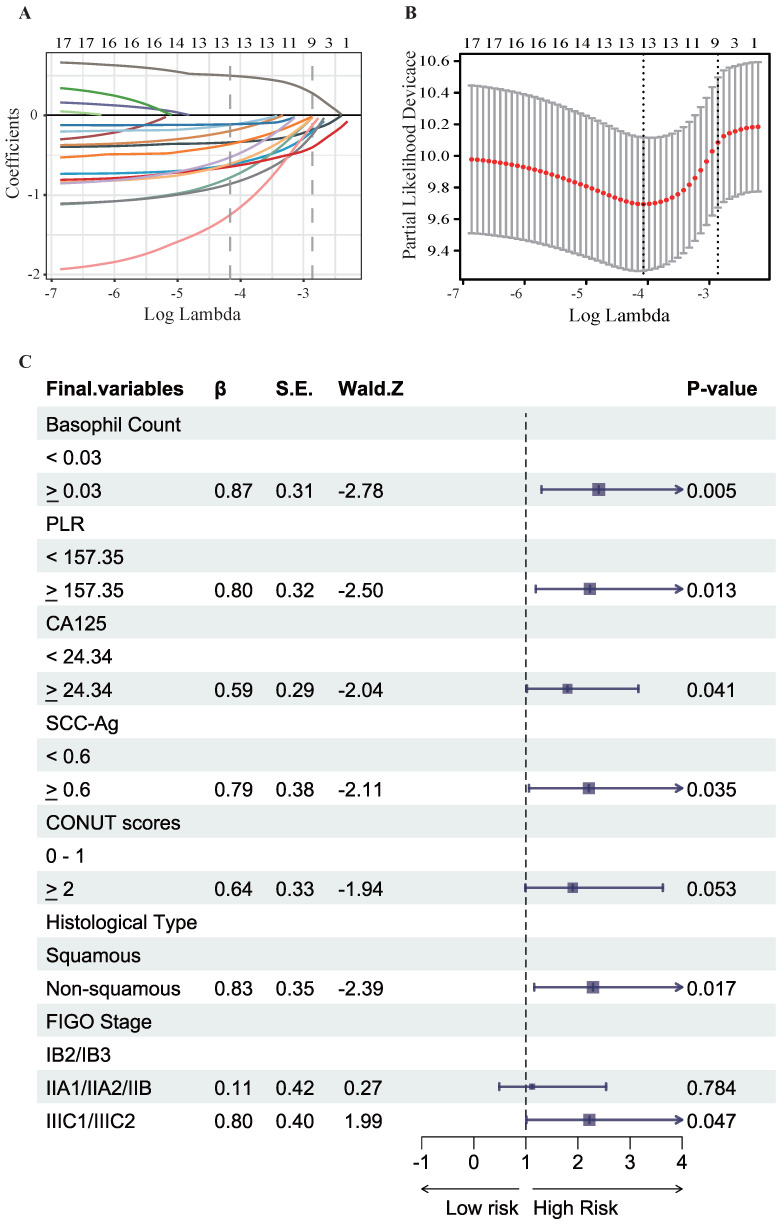
Results of LASSO regression and bidirectional stepwise multivariate Cox regression analysis for further selection of prognostic variables. (**A**) LASSO coefficient profiles for variables identified by Univariate Cox regression analysis, each coefficient profile plot is produced vs log (λ) sequence. (**B**) The partial likelihood binomial deviance is plotted vs log (λ). Thirteen variables with non-zero coefficients were selected by optimal lambda.min. (**C**) Results of bidirectional stepwise multivariate Cox regression analysis.

**Figure 4:**
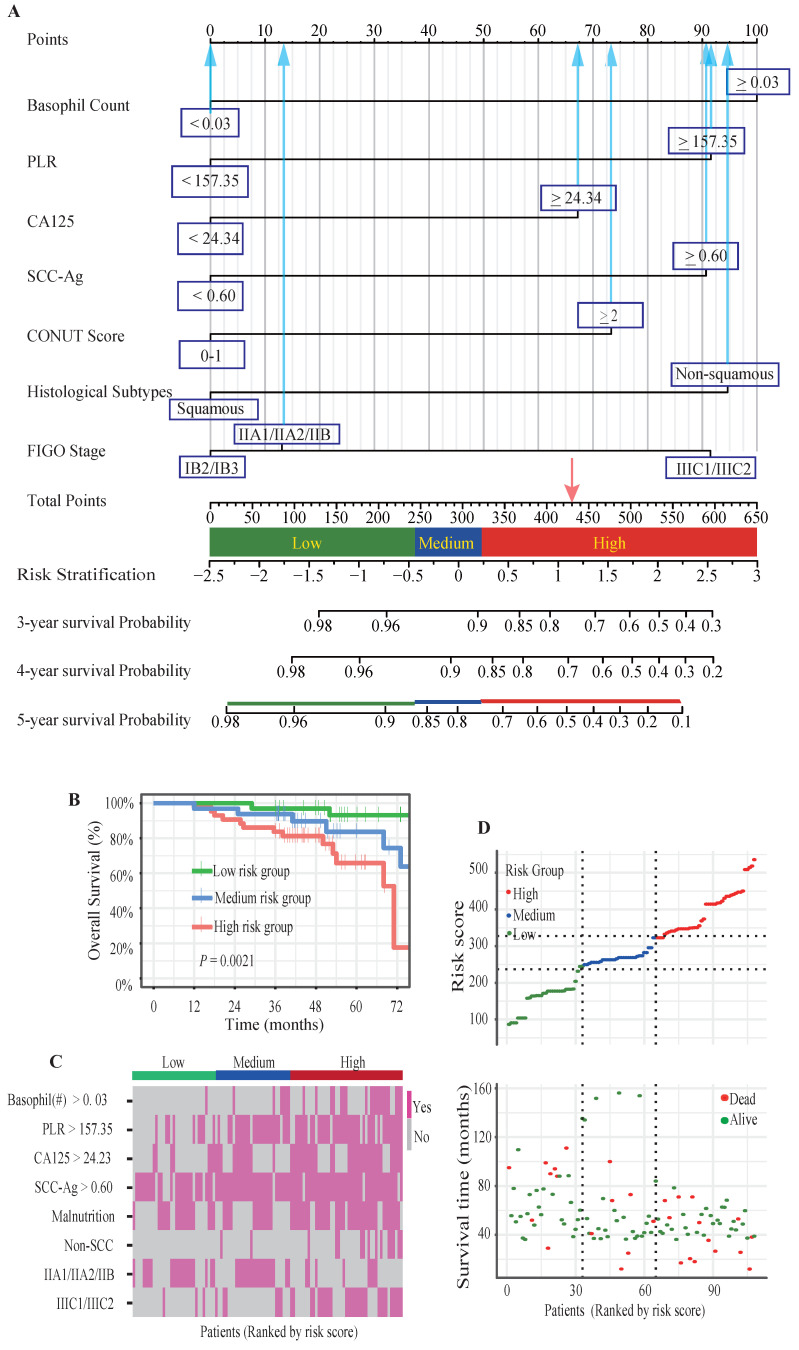
Nomogram model and risk stratification system. (**A**) The graph showed the nomogram for predicting 3-, 4- and 5-year OS of cervical cancer patients following postoperative radiotherapy. (**B**) Kaplan-Meier survival curves of three mortality risk subgroups in the validation cohort. (**C**) The heat map visualized the differential expression of risk factors across the three risk subgroups. (**D**) The distribution of risk scores and survival status of cervical cancer patients in the validation cohort.

To identify the most significant prognostic factors, we utilized a multi-step variable selection process as illustrated in Figure 2. In the training cohort, seventeen features were considered as potential prognostic factors (*P* < 0.20) from univariate Cox regression analysis (Table S1). The LASSO regression achieved its minimum penalized log-likelihood at the optimal tuning parameter λ of 0.017 (Figure 3). Thirteen variables with non-zero coefficients were identified by LASSO regression analysis (Figure 3A). Subsequently, these thirteen features were subjected to bidirectional stepwise multivariate Cox regression analysis which yielded seven optimal predictors with significant prognostic value: basophil count, PLR, CA125, SCC-Ag, CONUT score, histologic subtype, and FIGO stage (Figure 3C). Finally, a prognostic model based on the seven predictive factors was presented in the form of a visual nomogram to predict the 3-, 4-, and 5-year OS (Figure 4A). The proportional hazard assumptions of these variables and the entire model were further evaluated and no significant violations were detected (Figure S2). Additionally, the variance inflation factor (VIF) did not exceed 2, indicating no collinearity issues among the prognostic variables (Table S4).

A three-tier risk stratification system was constructed based on the prognostic model to facilitate the distinction of patients with different mortality risk levels. The nomogram was employed to calculate the total risk scores of each patient. To identify patients at differential risk of mortality, the "cutoff" package in R software was employed to determine optimal cut-off points (245 and 323) for stratifying patients into low-risk, intermediate-risk, and high-risk groups. Kaplan-Meier survival analysis with log-rank test revealed statistically significant differences in survival curves among all three risk groups (Figure 4B). The heatmaps intuitively illustrated the positive correlation between risk scores and expression of risk factors (Figure 4C). Additionally, a marked positive correlation was observed between patient survival outcomes and their corresponding risk scores (Figure 4D).

### Validation of the prognostic model

**Figure 5:**
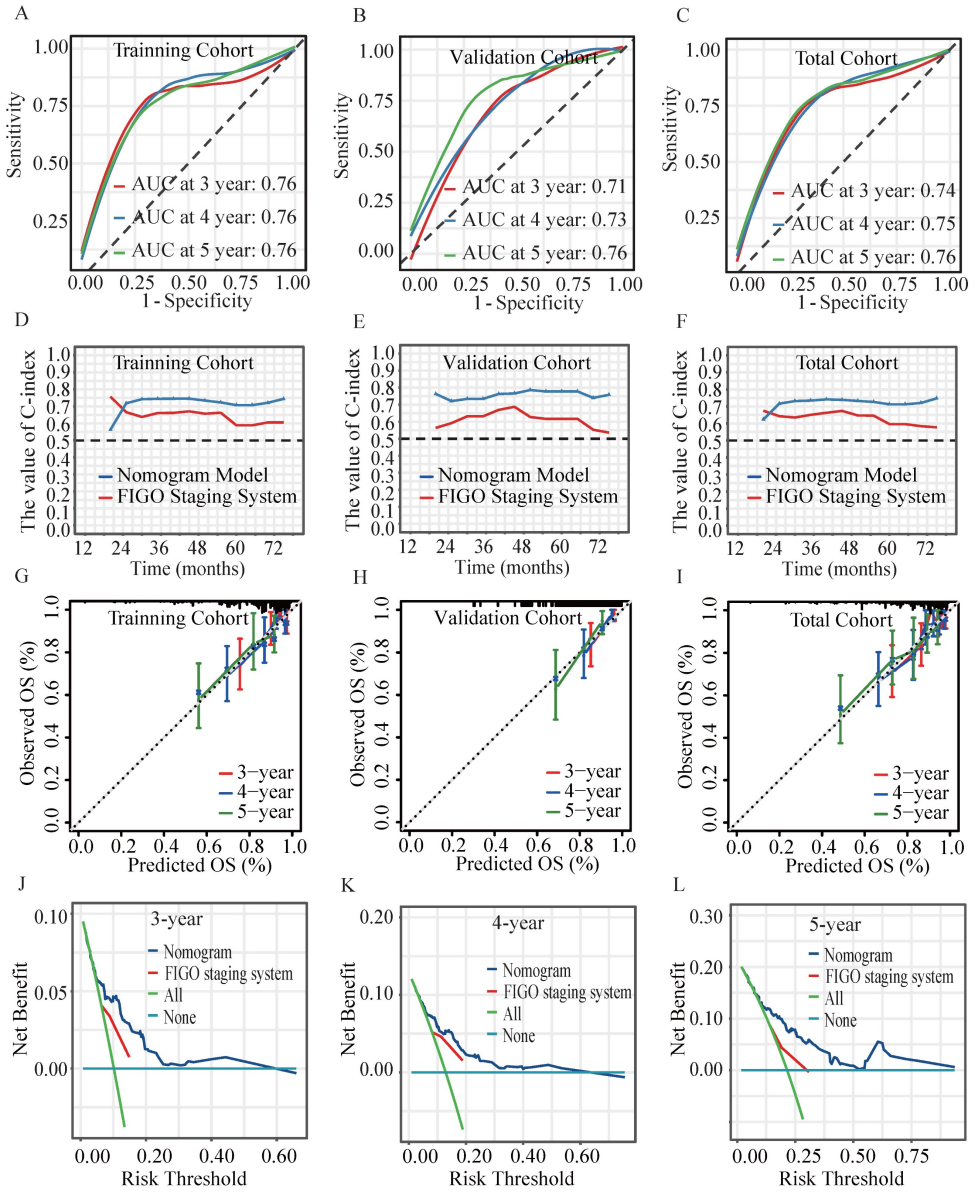
Verifying the performance of the prognostic model. The ROC curves of the nomogram model at 3-, 4- and 5-year in the (**A**) training cohort, (**B**) validation cohort and (**C**) total cohort. Time-dependent C-index values of the nomogram model and FIGO Staging System in (**D**) training cohort, (**E**) validation cohort and the (**F**) total cohort. The calibration curves of the nomogram model for predicting 3-, 4- and 5-year OS in (**G**) training cohort, (**H**) validation cohort and the (**I**) total cohort. The DCA for (**J**) 3-, (**K**) 4-, (**L**) 5-year OS prediction in total cohort.

In the validation cohort, the AUC for predicting OS at 3, 4, and 5 years achieved values of 0.71, 0.73 and 0.76, respectively (Figure 5A-5C). Time-dependent C-index analysis revealed that the nomogram significantly outperformed the sole FIGO staging system in predicting survival outcomes in all cohorts (Figure 5D-5F). Calibration curves displayed excellent concordance, indicating a strong correlation between the nomogram's predictions and observed OS (Figure 5G- 5I)). The DCA further substantiated the nomogram's clinical utility, which demonstrated a substantial net benefit in predicting 3-, 4-, and 5-year OS across a wide range of threshold probabilities (Figure 5J-5L)). Results of NRI and IDI analyses demonstrated the favorable applicability of the prognostic model (Table 2).

### Interrelationships among inflammatory levels, nutritional status, and clinicopathological features in cervical cancer patients underwent postoperative radiotherapy

**Figure 6:**
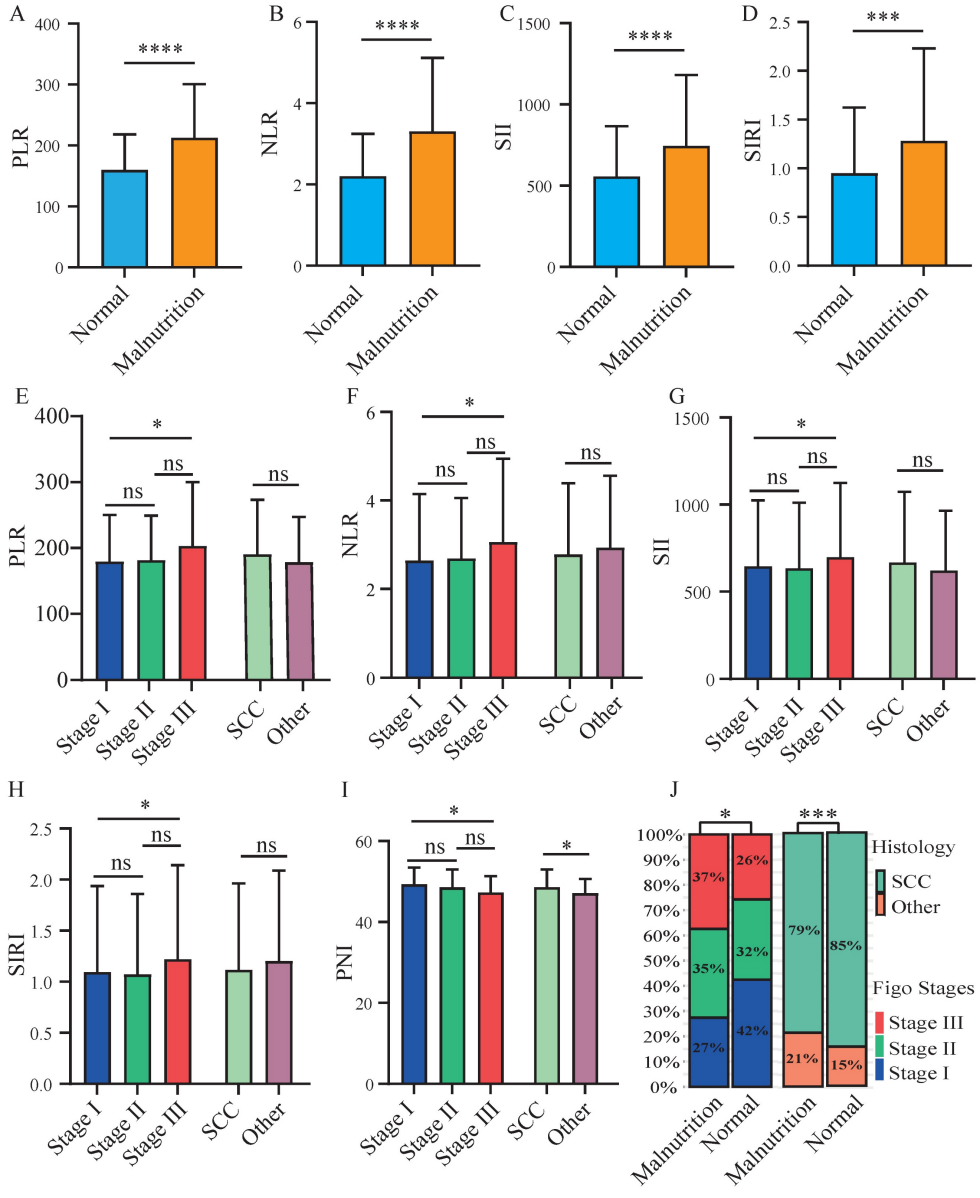
Correlations among inflammatory-nutritional indicators and clinicopathologic features. The figures illustrated the differential expression of inflammatory indicators in malnourished and well-nourished cervical cancer patients: (**A**) platelet-to-lymphocyte ratio (PLR), (**B**) neutrophil-to-lymphocyte ratio (NLR), (**C**) systemic immune inflammation index (SII), (**D**) system inflammation response index (SIRI). The figures illustrated the differential expression of inflammation-nutrition indicators in different FIGO stages and histological types: (**E**) PLR, (**F**) NLR, (**G**) SII, (**H**) SIRI, (**I**) PNI. (**J**) Distribution of FIGO stages and histological types between well-nourished and malnourished patients. P-values < 0.05 are considered statistically significant. *P < 0.05; **P < 0.01; ***P < 0.001, ****P < 0.0001.

A significant elevation of inflammatory indicators of PLR, NLR, SII, and SIRI was observed in patients categorized as mild or moderate malnutrition (CONUT scores > 2) (Figure 6A-6D). The results showed a higher prevalence of both advanced FIGO stages and non-squamous cell carcinoma in malnourished patients (Figure 6J). Patients with advanced FIGO stages exhibited significantly higher levels of PLR, NLR, SII, and SIRI compared to those with early FIGO stages (Figure 6E-6H). While a markedly lower of PNI was observed in patients with advanced FIGO stages or non-squamous cell carcinoma (Figure 6I). Furthermore, we examined the association between nutritional status and prognosis of patients across varying inflammatory levels. In the presence of elevated NLR and SIRI levels (Figure S3B, S3D), malnutritional patients exhibited a poorer prognosis. Similarly, for patients with low PLR levels (Figure S3E), malnutritional status was associated with a diminished OS. Malnutritional status was linked to a decreased OS in patients with both low and high SII levels (Figure S3C, S3G).

## Discussion

Cervical cancer patients with poorer nutrition (Figure 1A) or high inflammation (Figure 1B-1D) showed poorer prognosis compared to these with well nutrition and lower inflammatory levels. A positive correlation between poor nutritional status and elevated inflammatory indicators were observed (Figure 6A-6D). Patients with advanced FIGO-stages or non-squamous cell carcinoma exhibited elevated inflammatory and malnutritional indicators (Figure 6E-6I). Notably, after controlling inflammatory levels, malnutritional status remained a significant risk factor (Figure S3). Finally, the prognostic model and risk stratification system (Figure 4) which integrated the inflammatory and nutritional indicators of PLR, and CONUT score showed improved predictive ability.

There's quite a body of research had identified certain nutritional-inflammatory indicators as independent prognostic factors in cervical cancer^24^-^27^. However, heterogenous outcomes among these studies has been reported. Disparity in the study cohort size, difference in treatment, selection criteria for FIGO stages, and timing of data collection further contribute to these discrepancies. Additionally, the current problem is that most of these risk-analysis reports such as Guo J et al, Ferioli M et al and Ida N et al can provide information only on “which one is the risk factor”^25^, ^26^, ^28^. Nomograms have showed improved predictions and more individualized predictions compared with traditional staging systems in various malignancies including cervical cancer^29^-^31^. In earlier research, Wang et al. introduced a nomogram that incorporated the pretreatment levels of platelets and neutrophils, aiming to forecast the prognosis of cervical cancers patients undergoing radical radiotherapy^32^. Subsequently, Guo et al. developed a nomogram that included variables such as stages, BMI, NPR, PNI, SII, sarcopenia, and the intra-muscular adipose index to predict outcomes in cervical cancer^33^. Another investigation by Wang and colleagues revealed associations between PNI, GNRI, NLR, PLR, MLR, and survival in stage IIB-III cervical cancer patients receiving radiotherapy, leading to the construction of several nomograms based on these prognostic factors^24^. Nonetheless, the effectiveness and discriminative capacity of the nomogram by Guo et al. have not been thoroughly evaluated, and the consistency of Wang et al.'s nomogram was not tested^24^, ^33^. In addition, since the majority of the novel predictor factors will be of major value only if they add to the predictive value of traditional clinical and morphologic predictors. The clinical utility and added prognostic value of integrating nutritional-inflammatory indicators with traditional clinicopathological factors remain unexamined in published prognostic models. The comparative effectiveness of these nomograms to conventional staging systems, like the FIGO staging system, is unclear. Additionally, most of the nutritional-inflammatory prognostic factors were identified by using univariate and multivariate Cox regression analysis. Potential multicollinearity among nutritional-inflammatory indicators derived from hematological parameters, including PLR, NLR, SII, SIRI, and PNI, might compromise the reliability of a nomogram constructed solely using Cox regression^34^. Therefore, employing LASSO regression for selecting essential prognostic factors could offer a more suitable method for nomogram construction^35^.

Cervical cancer patients who received postoperative adjuvant radiotherapy exhibited a higher incidence of treatment-related adverse events, including bowel dysfunction, urinary complications, and fatigue, compared to those receiving radiotherapy alone^36^. Concurrently, tumor-induced metabolic disturbances exacerbated these effects, predisposing patients to malnutrition, which may impact treatment efficacy and overall prognosis^10^. However, the impact of pretreatment nutritional-inflammatory status on the long-term prognosis of patients receiving postoperative adjuvant radiotherapy remains inadequately explored. Our study focused exclusively on patients who received postoperative radiotherapy. In the current study, LASSO regression and stepwise regression were employed to identify key predictors while mitigating the influence of multicollinearity among variables (Figure 3, Figure S2, Table S4). In our study, a novel prognostic model incorporating both nutritional and inflammatory indicators was developed, which uniquely included basophil count and CA125 as predictive factors (Figure 4A). The nomogram incorporating nutritional-inflammatory factors demonstrated superior predictive accuracy for long-term patient outcomes compared to the traditional FIGO staging system (Figure 5, Table 2). Furthermore, our prognostic model was superior to the models of Wang HB et al and Wang H et al (Figure S4, Table S5)^24^, ^32^. However, the observed differences might be influenced by variations in patient enrollment criteria and treatment regimens. The findings of this study implied that a combined therapeutic approach targeting nutritional improvement and inflammation reduction prior to radiotherapy held promise for improving the overall survival of cervical cancer patients requiring postoperative adjuvant radiotherapy.

Tumor-associated inflammatory contributed to the impaired immune function and treatment resistance^37^, ^38^. Despite the hematological-derived inflammatory biomarkers may provide an incomplete picture of systemic inflammation response in cancer patients, potentially leading to inaccurate classification of inflammatory status. Systemic inflammatory biomarkers such as PLR, SII and SIRI offered advantages due to their simplicity, cost-effectiveness, and practicality. These inflammatory biomarkers were gaining increasing attention for their potential role in assessing prognosis and guiding treatment decisions in multiple cancers including cervical cancer. Consistent with published researches^8^, ^39^, we found elevated inflammatory indicators of PLR, NLR, and SII were independent risk factors in CC (Figure 1). Platelet-derived cytokines, such as VEGF, have been shown to be critical drivers of tumor development^39^. Additionally, tumor-induced inflammatory cytokines, such as interleukin-6 (IL-6), had been implicated as key drivers of elevated platelet counts observed in cervical and ovarian cancer patients^18^, ^40^. This interplay may establish a vicious cycle, further accelerating tumor progression. Moreover, our study unveiled the potential of basophil as a novel prognostic biomarker in CC (Figure 3C). Despite their relatively low abundance, basophils induced immune mediators such as VEGF-A/B and histamine were implicated in tumor progression^41^. However, the underlying mechanisms and specific role of basophil in CC remained further investigation.

Multiple studies have indicated the high prevalence of malnutrition in CC particularly for patients receiving postoperative radiotherapy^8^, ^36^. In our study, 54.8% (CONUT > 2) and 20.3% (BMI < 18.5 kg/m2) of CC patients were classified as mildly or moderately malnourished before receiving radiotherapy, respectively (Table S1). Notably, high levels of inflammation had been well-established as a potent driver of malnutrition, leading to a substantial deterioration in their nutritional status^7^. Previous studies have demonstrated that elevated levels of pro-inflammatory cytokines, including IL-1β, IL-6, and TNF-α, played a significant role in the pathogenesis of cancer-associated cachexia^14^, ^15^. Malnourished patients with gynecological cancers suffered significantly poorer prognosis compared to well-nourished patients, even after adjusting the high-risk tumor characteristics^6^. Pervious researches had highlighted a critical challenge in cancer care for the diminished responsiveness to nutritional support in patients with high levels of inflammation^15^. Our study further revealed that after controlling the baseline inflammatory levels, patients with well nutritional status still experienced a better prognosis compared to these with poor nutritional state (Figure S3). These findings highlighted the imperative for a multifactorial approach to managing cancer-associated malnutrition. A multi-targeted therapeutic strategy targeting on the concurrent modulation of inflammatory and nutritional status held promise for improved prognosis of patients with CC.

The current study possessed inherent limitations that necessitated further research. The retrospective design and relatively modest sample size restricted the generalizability of our findings. While we endeavored to incorporate a comprehensive set of relevant variables, some inflammatory and nutritional biomarkers associated with prognosis remained unattainable. Moreover, the absence of external validation by another institution limited the generalizability of our proposed prognostic model. Further prospective research is warranted to validate the effectiveness of combined nutritional and inflammatory interventions in improving patient quality of life and prognosis.

## Conclusions

The current study unveiled the poorer nutritional and high inflammatory status collectively contributed to the poorer prognosis of patients with cervical cancer who received the adjuvant radiotherapy. Patients with poorer nutritional status exhibited higher levels of inflammation, particularly those in advanced clinical stages and with non-squamous cell carcinoma. The prognostic model incorporating both inflammatory and nutritional parameters held promise for enhancing the prediction of overall survival in patients receiving postoperative radiotherapy.

## Supplementary Material

Supplementary figures and tables.

## Figures and Tables

**Table 1:** Clinical characteristics of the patients.

Patient Characteristics	Number	Percentage (%)
**Age**		
Median age	50 (range 26-76)	
≤ 45	141	43.4%
> 45	184	56.6%
KPS		
≤ 70	5	1.5%
> 70	320	98.5%
**FIGO Stage**		
Stage IB2	76	23.4%
Stage IB3	23	7.1%
Stage IIA1	56	17.2%
Stage IIA2	46	14.2%
Stage IIB	10	3.1%
Stage IIIC1	103	31.7%
Stage IIIC2	11	3.4%
**Histology**		
Squamous Cell carcinoma	271	83.4%
Adenocarcinoma	29	8.9%
Other	25	7.7%
**Lymph node status**		
No	211	64.9%
Yes	114	35.1%

***Abbreviations:*** KPS, Karnofsky performance status; FIGO, International Federation of Gynecology and Oncology

**Table 2:** NRI and IDI of the nomogram in survival prediction compared with FIGO staging system.

Index	NRI			IDI	
	Estimate (95% CI)	*P*-value		Estimate (95% CI)	*P*-value
**Training group**					
For 3-year OS	0.37 (0.06-0.55)	0.016		0.10 (0.03-0.25)	<0.001
For 4-year OS	0.26 (0.10-0.52)	0.008		0.10 (0.03-0.26)	<0.001
For 5-year OS	0.34 (0.19-0.62)	<0.001		0.16 (0.08-0.33)	<0.001
**Validation group**					
For 3-year OS	0.31 (-0.07-0.61)	0.132		0.06 (0.00-0.32)	0.044
For 4-year OS	0.42 (0.06-0.63)	0.016		0.08 (0.02-0.32)	0.004
For 5-year OS	0.50 (0.17-0.73)	<0.001		0.17 (0.07-0.37)	<0.001
**Full group**					
For 3-year OS	0.30 (0.07-0.41)	0.004		0.08 (0.03-0.18)	<0.001
For 4-year OS	0.22 (0.18-0.46)	<0.001		0.08 (0.03-0.19)	<0.001
For 5-year OS	0.40 (0.23-0.60)	<0.001		0.15 (0.08-0.27)	<0.001

## References

[1] Bray F, Laversanne M, Sung H, Ferlay J, Siegel RL, Soerjomataram I (2024). Global cancer statistics 2022: GLOBOCAN estimates of incidence and mortality worldwide for 36 cancers in 185 countries. CA Cancer J Clin.

[2] Lee SJ, Kim M, Kwak YK, Kang HJ (2024). The impact of boost radiation therapy after hysterectomy on cervical cancer patients with close or positive resection margins. Clin Transl Oncol.

[3] Kim H, Cho WK, Kim YJ, Kim YS, Park W (2020). Significance of the number of high-risk factors in patients with cervical cancer treated with radical hysterectomy and concurrent chemoradiotherapy. Gynecol Oncol.

[4] Wright JD, Matsuo K, Huang Y, Tergas AI, Hou JY, Khoury-Collado F (2019). Prognostic Performance of the 2018 International Federation of Gynecology and Obstetrics Cervical Cancer Staging Guidelines. Obstet Gynecol.

[5] Kumar A, Gurram L, Naga Ch P, Nayak P, Mulye G, Chopra S (2024). Correlation of Hematological Parameters With Clinical Outcomes in Cervical Cancer Patients Treated With Radical Radio(chemo)therapy: A Retrospective Study. Int J Radiat Oncol Biol Phys.

[6] Morton M, Patterson J, Sciuva J, Perni J, Backes F, Nagel C (2023). Malnutrition, sarcopenia, and cancer cachexia in gynecologic cancer. Gynecol Oncol.

[7] Arends J, Bachmann P, Baracos V, Barthelemy N, Bertz H, Bozzetti F (2017). ESPEN guidelines on nutrition in cancer patients. Clin Nutr.

[8] Goins EC, Weber JM, Truong T, Moss HA, Previs RA, Davidson BA (2022). Malnutrition as a risk factor for post-operative morbidity in gynecologic cancer: Analysis using a national surgical outcomes database. Gynecol Oncol.

[9] Argefa TG, Roets L (2022). Malnutrition and the Survival of Cervical Cancer Patients: A Prospective Cohort Study Using the PG-SGA Tool. Nutr Cancer.

[10] Laan J, van Lonkhuijzen L, Hinnen K, Pieters B, Dekker I, Stalpers L (2024). Malnutrition is associated with poor survival in women receiving radiotherapy for cervical cancer. Int J Gynecol Cancer.

[11] Bharadwaj S, Ginoya S, Tandon P, Gohel TD, Guirguis J, Vallabh H (2016). Malnutrition: laboratory markers vs nutritional assessment. Gastroenterol Rep (Oxf).

[12] Zhang G, Zhang Y, He F, Wu H, Wang C, Fu C (2021). Preoperative controlling nutritional status (CONUT) score is a prognostic factor for early-stage cervical cancer patients with high-risk factors. Gynecol Oncol.

[13] Niu Z, Yan B (2023). Prognostic and clinicopathological effect of the prognostic nutritional index (PNI) in patients with cervical cancer: a meta-analysis. Ann Med.

[14] Grivennikov SI, Greten FR, Karin M (2010). Immunity, inflammation, and cancer. Cell.

[15] Stumpf F, Keller B, Gressies C, Schuetz P (2023). Inflammation and Nutrition: Friend or Foe?. Nutrients.

[16] Jiang S, Liu J, Chen X, Zheng X, Ruan J, Ye A (2019). Platelet-lymphocyte ratio as a potential prognostic factor in gynecologic cancers: a meta-analysis. Arch Gynecol Obstet.

[17] Templeton AJ, Ace O, McNamara MG, Al-Mubarak M, Vera-Badillo FE, Hermanns T (2014). Prognostic role of platelet to lymphocyte ratio in solid tumors: a systematic review and meta-analysis. Cancer Epidemiol Biomarkers Prev.

[18] Domenici L, Tonacci A, Aretini P, Garibaldi S, Perutelli A, Bottone P (2021). Inflammatory Biomarkers as Promising Predictors of Prognosis in Cervical Cancer Patients. Oncology.

[19] Chao B, Ju X, Zhang L, Xu X, Zhao Y (2020). A Novel Prognostic Marker Systemic Inflammation Response Index (SIRI) for Operable Cervical Cancer Patients. Front Oncol.

[20] Huang H, Liu Q, Zhu L, Zhang Y, Lu X, Wu Y (2019). Prognostic Value of Preoperative Systemic Immune-Inflammation Index in Patients with Cervical Cancer. Sci Rep.

[21] Mizunuma M, Yokoyama Y, Futagami M, Aoki M, Takai Y, Mizunuma H (2015). The pretreatment neutrophil-to-lymphocyte ratio predicts therapeutic response to radiation therapy and concurrent chemoradiation therapy in uterine cervical cancer. Int J Clin Oncol.

[22] Chen L, Zhang F, Sheng XG, Zhang SQ (2015). Decreased pretreatment lymphocyte/monocyte ratio is associated with poor prognosis in stage Ib1-IIa cervical cancer patients who undergo radical surgery. Onco Targets Ther.

[23] Ignacio de Ulíbarri J, González-Madroño A, de Villar NG, González P, González B, Mancha A (2005). CONUT: a tool for controlling nutritional status. First validation in a hospital population. Nutr Hosp.

[24] Wang HB, Xu XT, Tian MX, Ding CC, Tang J, Qian Y (2023). Prognostic values of the prognostic nutritional index, geriatric nutritional risk index, and systemic inflammatory indexes in patients with stage IIB-III cervical cancer receiving radiotherapy. Front Nutr.

[25] Guo J, Lv W, Wang Z, Shang Y, Yang F, Zhang X (2023). Prognostic Value of Inflammatory and Nutritional Markers for Patients With Early-Stage Poorly-to Moderately-Differentiated Cervical Squamous Cell Carcinoma. Cancer Control.

[26] Ferioli M, Benini A, Malizia C, Forlani L, Medici F, Laghi V (2023). Classical Prognostic Factors Predict Prognosis Better than Inflammatory Indices in Locally Advanced Cervical Cancer: Results of a Comprehensive Observational Study including Tumor-, Patient-, and Treatment-Related Data (ESTHER Study). J Pers Med.

[27] He X, Li JP, Liu XH, Zhang JP, Zeng QY, Chen H (2018). Prognostic value of C-reactive protein/albumin ratio in predicting overall survival of Chinese cervical cancer patients overall survival: comparison among various inflammation based factors. J Cancer.

[28] Ida N, Nakamura K, Saijo M, Kusumoto T, Masuyama H (2018). Prognostic nutritional index as a predictor of survival in patients with recurrent cervical cancer. Mol Clin Oncol.

[29] Seo Y, Yoo SY, Kim MS, Yang KM, Yoo HJ, Kim JH (2011). Nomogram prediction of overall survival after curative irradiation for uterine cervical cancer. Int J Radiat Oncol Biol Phys.

[30] Shim SH, Lee SW, Park JY, Kim YS, Kim DY, Kim JH (2013). Risk assessment model for overall survival in patients with locally advanced cervical cancer treated with definitive concurrent chemoradiotherapy. Gynecol Oncol.

[31] Rose PG, Java J, Whitney CW, Stehman FB, Lanciano R, Thomas GM (2015). Nomograms Predicting Progression-Free Survival, Overall Survival, and Pelvic Recurrence in Locally Advanced Cervical Cancer Developed From an Analysis of Identifiable Prognostic Factors in Patients From NRG Oncology/Gynecologic Oncology Group Randomized Trials of Chemoradiotherapy. J Clin Oncol.

[32] Wang H, Chen WM, Zhou YH, Shi JP, Huang YQ, Wang WJ (2020). Combined PLT and NE to predict the prognosis of patients with locally advanced cervical cancer. Sci Rep.

[33] Guo H, Feng S, Li Z, Yin Y, Lin X, Yuan L (2023). Prognostic Value of Body Composition and Systemic Inflammatory Markers in Patients with Locally Advanced Cervical Cancer Following Chemoradiotherapy. J Inflamm Res.

[34] Vatcheva KP, Lee M, McCormick JB, Rahbar MH (2016). Multicollinearity in Regression Analyses Conducted in Epidemiologic Studies. Epidemiology (Sunnyvale).

[35] Mueller-Using S, Feldt T, Sarfo FS, Eberhardt KA (2016). Factors associated with performing tuberculosis screening of HIV-positive patients in Ghana: LASSO-based predictor selection in a large public health data set. BMC Public Health.

[36] Liu YM, Ni LQ, Wang SS, Lv QL, Chen WJ, Ying SP (2018). Outcome and prognostic factors in cervical cancer patients treated with surgery and concurrent chemoradiotherapy: a retrospective study. World J Surg Oncol.

[37] Balkwill F, Mantovani A (2001). Inflammation and cancer: back to Virchow?. Lancet.

[38] Zappavigna S, Cossu AM, Grimaldi A, Bocchetti M, Ferraro GA, Nicoletti GF (2020). Anti-Inflammatory Drugs as Anticancer Agents. Int J Mol Sci.

[39] Holub K, Biete A (2019). Impact of systemic inflammation biomarkers on the survival outcomes of cervical cancer patients. Clin Transl Oncol.

[40] Stone RL, Nick AM, McNeish IA, Balkwill F, Han HD, Bottsford-Miller J (2012). Paraneoplastic thrombocytosis in ovarian cancer. New Engl J Med.

[41] Chauhan J, Stavraka C, Grandits M, Palhares L, Josephs DH, Lacy KE (2022). Clinical and Translational Significance of Basophils in Patients with Cancer. Cells.

