# Supplementary material for: A survival nomogram involving nutritional-inflammatory indicators for cervical cancer patients receiving adjuvant radiotherapy
Source: J Cancer. 2024 Sep 9;15(17):5773–85. doi: 10.7150/jca.100564 (PMC11414624; doi:10.7150/jca.100564)
Supplement: Supplementary file 1 — Supplementary figures and tables. [file jcav15p5773s1.pdf]

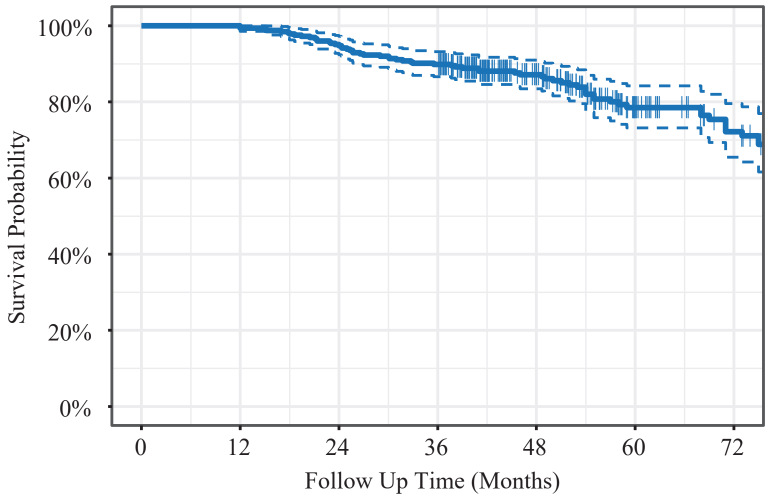

Figure S1. Overall survival rate of the study population. This study ultimately comprised 325 cervical cancer patients underwent postoperative radiotherapy. With a median follow-up of 50.5 months, the 3-, 4-, and 5-year overall survival rates was 89.8%, 87.1%, and 78.5%, respectively.

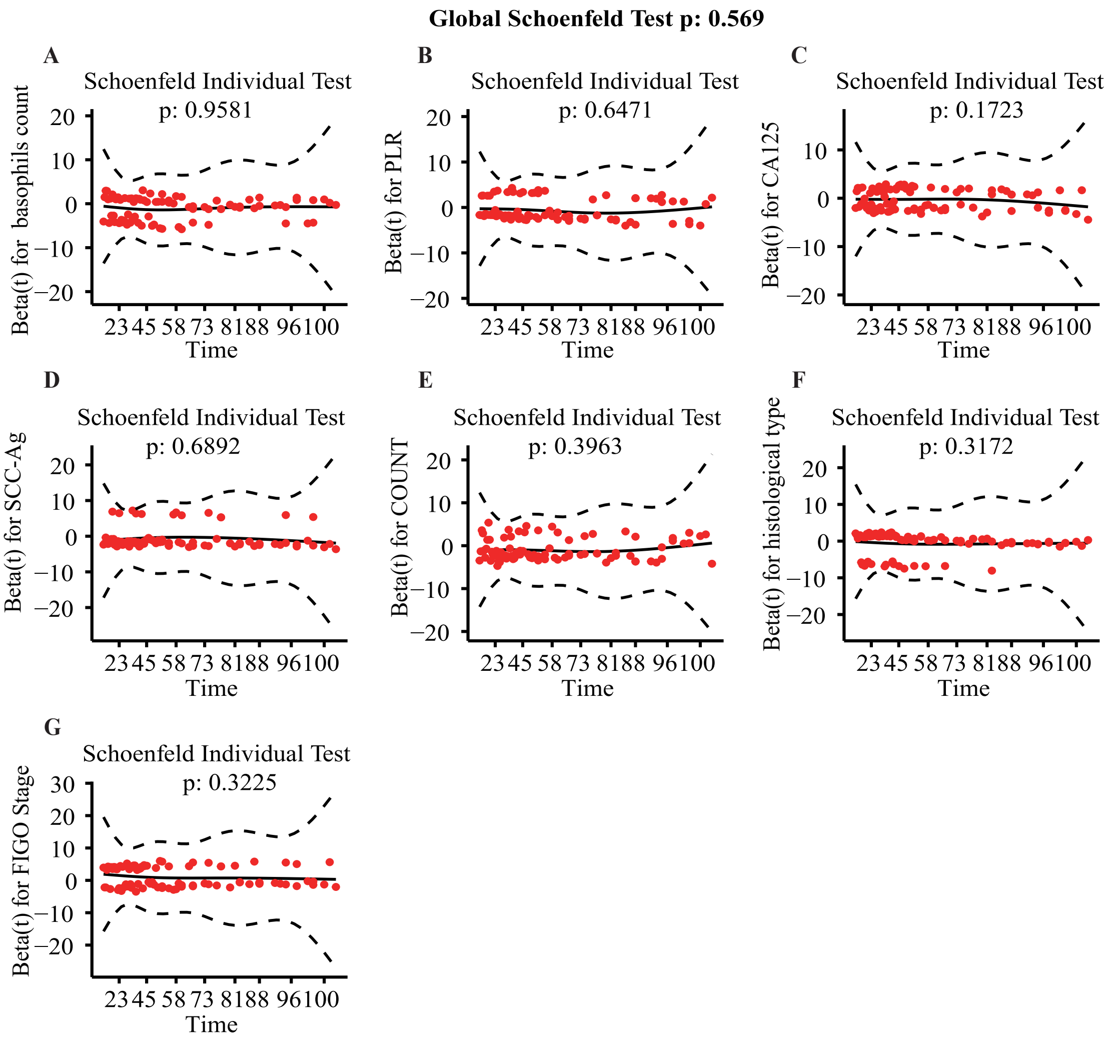

Figure S2. Plots of the scaled Schoenfeld residuals versus time for the Cox proportional hazards model fit to the total cohort. The solid line (bt) gives the estimated effect of the predictors through time in the experiment (with confidence intervals). Plots are given for the predictors: (A) Absolute count of basophil cells, (B) PLR, (C) CONUT score, (D) CA125, (E) SCC-Ag, (F) Histological subtypes, and (G) FIGO-Stage. No significant violations of the assumption of proportional hazards were found for the predictors.

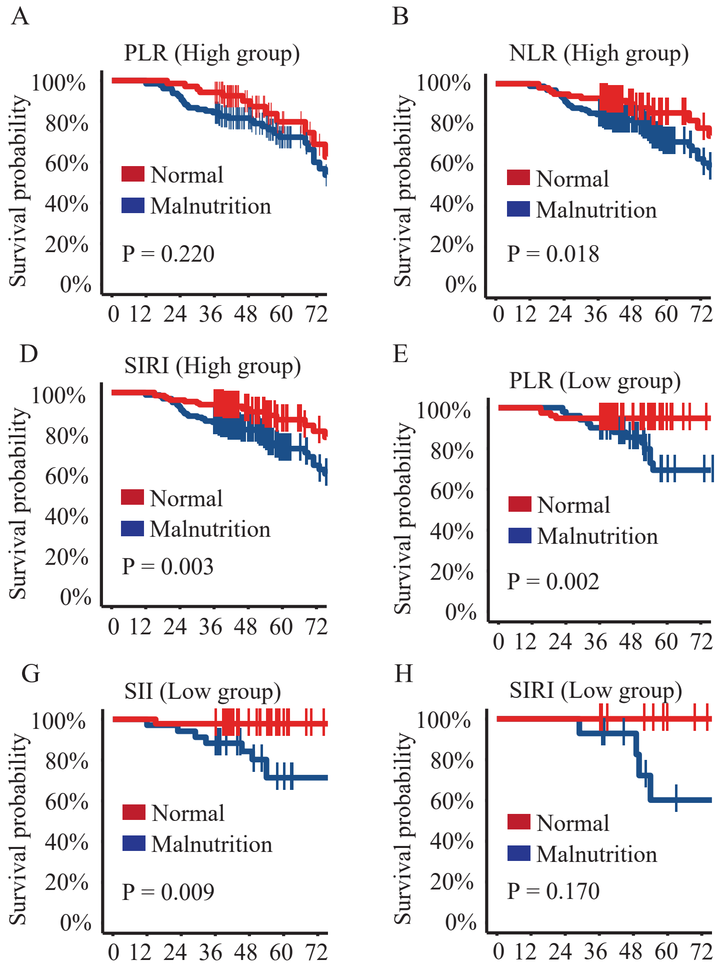

Figure S3. Impact of nutritional status on overall survival stratified by inflammatory index levels. Impact of nutritional status on overall survival in patients with high inflammatory indices: (A) PLR, (B) NLR, (C) SII, (D) SIRI. Impact of nutritional status on overall survival in patients with low inflammatory indices: (E) PLR, (F) NLR, (G) SII, (H) SIRI. PLR, platelet-to-lymphocyte; NLR, neutrophil-to-lymphocyte ratio; SII, systemic immune inflammation index; SIRI, system inflammation response index;

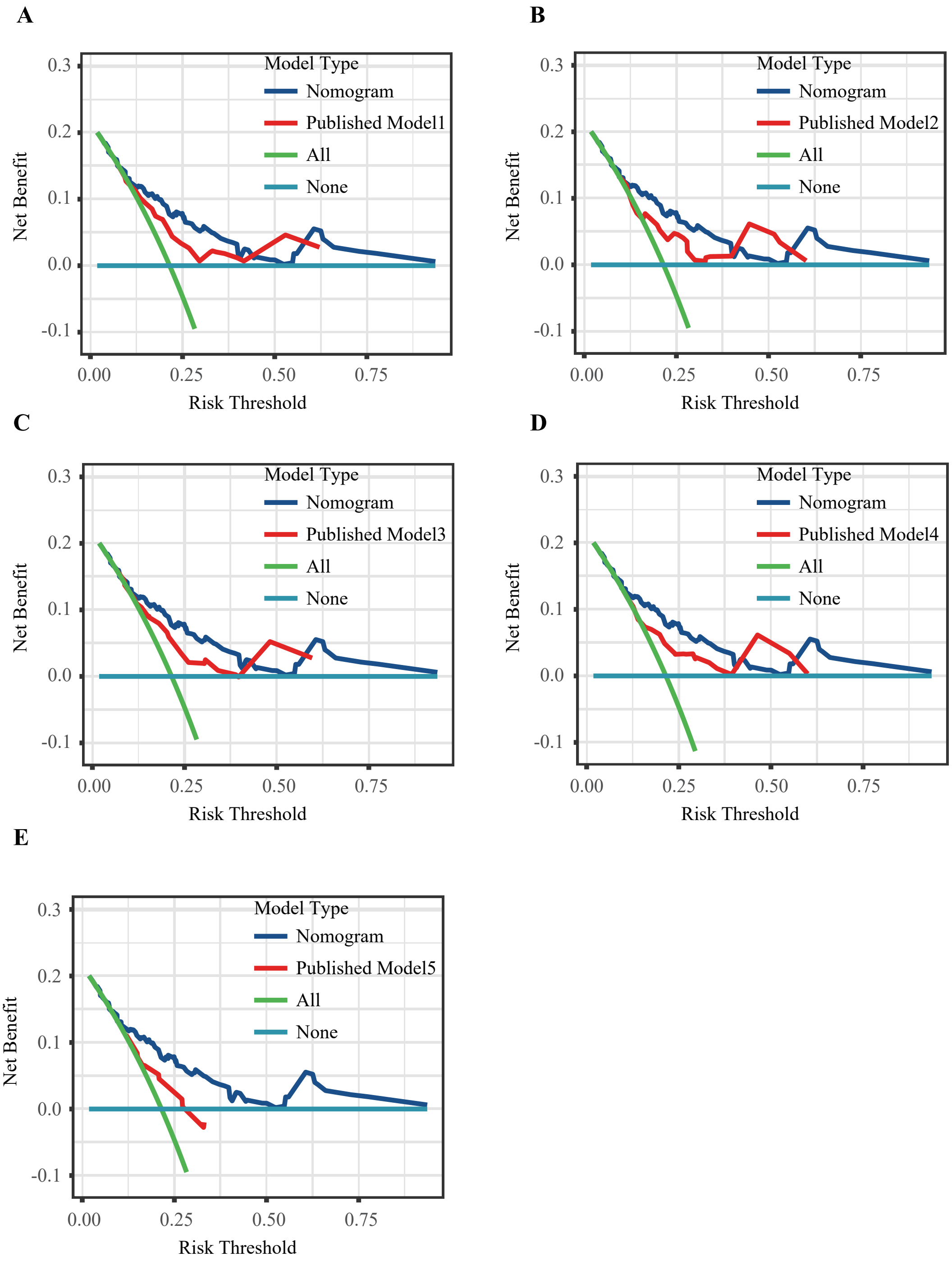

Figure S4. Comparison of decision curve analysis (DCA) between the developed nomogram and established models. The x-axis represents the risk threshold, and the y-axis denotes the net benefit. The blue line visualizes the performance of the developed nomogram, while the red line illustrates the performance of established models. (**A**) Developed nomogram vs. established model1; (**B**) Developed nomogram vs. established model2; (**C**) Developed nomogram vs. established model3; (**D**) Developed nomogram vs. established model4; (**E**) Developed nomogram vs. established model5; Prognostic indicators of age, histological type, FIGO staging system and PLR were introduced into model1[24]. Prognostic indicators of age, histological type, FIGO staging system and MLR were introduced into model2[24]. Prognostic indicators of age, histological type, FIGO staging system and NLR were introduced into model3[24]. Prognostic indicators of age, histological type, FIGO staging system and PNI were introduced into model4[24]. Prognostic indicators of FIGO staging system, platelet count and neutrophils count were introduced into model5[32].

Variables	Univariate Cox regression analysis		Log-Rank survival analysis
	HR (% 95 CI)	*P*-value	*P*-value

Table S1. Results of univariate Cox regression and Log-Rank survival analyses.

**Variables**	**Univariate Cox regression analysis**		**Log-Rank survival analysis**
	**HR (% 95 CI)**	***P*-value**	***P*-value**
**Age**			0.182
≤ 45	Reference		
> 45	1.30 (0.40 - 4.17)	0.664	
**Histological Type**			0.011
Squamous Cell carcinoma	Reference		
Other	2.30 (1.18 – 4.49)	0.015	
**FIGO Stage**			0.005
IB2/IB3	Reference		
IIA1/IIA2/IIB	1.62 (0.74 - 3.55)	0.128	
IIIC1/IIIC2	2.89 (1.35 – 6.17)	0.006	
**CEA**			0.141
< 2.17	Reference		
≥ 2.17	1.27 (0.73-2.20)	0.396	
**CA125**			0.034
< 24.34	Reference		
≥ 24.34	2.10 (1.22-3.63)	0.007	
**CA153**			0.039
< 9.80	Reference		
≥ 9.80	1.48 (0.85-2.60)	0.169	
**CA199**			0.429
< 8.20	Reference		
≥ 8.20	0.99 (0.57-1.72)	0.977	
**SCC-Ag**			0.148
< 0.6	Reference		
≥ 0.6	1.67 (0.82-3.40)	0.159	
**Total Protein (g/L)**			0.984
< 69.80	Reference		
≥ 69.80	0.79 (0.44-1.43)	0.436	
**Albumin (g/L)**			0.921
< 43.10	Reference		
≥ 43.10	0.95 (0.55-1.65)	0.860	
**Prealbumin (mg/dL)**			0.824
< 248.00	Reference		

Variables	Univariate Cox regression analysis		Log-Rank survival analysis
	HR (% 95 CI)	*P*-value	*P*-value
≥ 248.00	1.08 (0.64-1.82)	0.785	
**CREA (μmol/L)**			0.548
< 57.00	Reference		
≥ 57.00	0.81 (0.48-1.38)	0.446	
**RBP (mg/L)**			0.856
< 44.00	Reference		
≥ 44.00	0.96 (0.55-1.67)	0.881	
**TC (mmol/L)**			0.544
< 210.64	Reference		
≥ 210.64	1.58 (0.90-2.80)	0.114	
**BMI (kg/m²)**			
Healthy-weight	Reference		0.690
Under-weight	0.63 (0.30-1.34)	0.132	
Obesity	0.96 (0.34-2.69)	0.941	
**CONUT scores**			0.001
0 - 1	Reference		
≥ 2	1.72 (1.01-2.95)	0.047	
**PNI**			0.770
< 51.22	Reference		
≥ 51.22	0.94 (0.53-1.64)	0.818	
**PLR**			0.003
< 157.35	Reference		
≥ 157.35	2.49 (1.39-4.46)	0.002	
**NLR**			0.046
< 1.67	Reference		
≥ 1.67	1.71 (0.81-3.61)	0.161	
**MLR**			0.619
< 3.58	Reference		
≥ 3.58	1.08 (0.64-1.83)	0.777	
**SII**			0.005
< 385.80	Reference		
≥ 385.80	2.50 (1.18-5.30)	0.016	
**SIRI**			0.651
< 0.36	Reference		
≥ 0.36	1.48 (0.53-4.09)	0.455	
**WBC (*10^9/L)**			0.919
< 5.90	Reference		
≥ 5.90	1.30 (0.40 - 4.17)	0.664	
**RBC (*10^12/L)**			0.290

Variables	Univariate Cox regression analysis		Log-Rank survival analysis
	HR (% 95 CI)	*P*-value	*P*-value
< 3.67	Reference		
≥ 3.67	1.84 (0.66 -5.10)	0.241	
**HGB (g/L)**			0.782
< 110	Reference		
≥ 110	1.11 (0.62-1.97)	0. 725	
**PLT (*10^9/L)**			0.305
< 202	Reference		
≥ 202	1.65 (0.92-2.98)	0.094	
**Lymphocyte (*10^9/L)**			0.305
< 1.03	Reference		
≥ 1.03	0.79 (0.31-1.99)	0.615	
**Monocyte (*10^9/L)**			0.521
< 0.49	Reference		
≥ 0.49	0.70 (0.17-2.90)	0.626	
**Neutrophil (*10^9/L)**			0.712
< 2.14	Reference		
≥ 2.14	1.33 (0.57-3.09)	0.515	
**Basophil (*10^9/L)**			0.001
< 0.03	Reference		
≥ 0.03	1.90 (1.10-3.27)	0.022	
**Eosinophil (*10^9/L)**			0.060
< 0.02	Reference		
≥ 0.02	1.45 (0.66-3.22)	0.356	
**Lymphocyte (%)**			0.210
< 16.3	Reference		
≥ 16.3	2.41 (0.75-7.71)	0.139	
**Monocyte (%)**			0.513
< 5.5	Reference		
≥ 5.4	1.47 (0.78-2.80)	0.237	
**Neutrophil (%)**			0.932
< 56.8	Reference		
≥ 56.8	1.03 (0.57-1.86)	0.915	
**Basophil (%)**			0.624
< 0.40	Reference		
≥ 0.40	1.19 (0.69-2.05)	0.528	
**Eosinophil (%)**			0.963
< 4.80	Reference		
≥ 4.80	1.29 (0.58-2.87)	0.534	
**MCV (fl)**			0.043

Variables	Univariate Cox regression analysis		Log-Rank survival analysis
	HR (% 95 CI)	*P*-value	*P*-value
< **91.5**	Reference		
≥ 91.5	1.58 (0.94-2.65)	0.083	
**MCH (pg)**			0.028
< 30	Reference		
≥ 30	1.45 (0.87-2.44)	0.158	
**MPV (%)**			0.796
< 10.70	Reference		
≥ 10.70	0.91 (0.54-1.54)	0.724	
**PDW (%)**			0.691
< 12.30	Reference		
≥ 12.30	0.87 (0.51-1.47)	0.601	
**RDW (%)**			0.490
< 14.10	Reference		
≥ 14.10	1.15 (0.68-1.96)	0.598	
**PCT (%)**			0.284
< 0.22	Reference		
≥ 0.22	1.62 (0.93-2.84)	0.090	

Abbreviations: %, percentage; FIGO, International Federation of Gynecology and Oncology; CEA, carcinoembryonic antigen; CA125, cancer antigen 125; CA153, cancer antigen 153; CA199, cancer antigen 199; SCC-Ag, squamous cell carcinoma antigen; CREA, serum creatinine; RBP, retinol binding protein; TC, total cholesterol; MBI, body mass index; CONUT, controlling nutritional status; PNI, prognostic nutritional index; PLR, platelet-to-lymphocyte; NLR, neutrophil-to-lymphocyte ratio; MLR, monocyte -to- lymphocyte; SII, systemic immune inflammation index; SIRI, system inflammation response index; WBC, white blood cell count; RBC, red blood cell count; HGB, hemoglobin concentration; PLT, platelet count; MCV, mean corpuscular volume; MCH, mean corpuscular hemoglobin; MPV, mean platelet volume; PDW, platelet distribution width; RDW, red cell distribution width; PCT, platelet hematocrit;

Nutritional-Inflammatory Indicators	Total N=325 (%)	Training cohort N=217 (%)	Validation cohort N=108 (%)	*P* -value

Table S2. Baseline levels of nutritional-inflammatory indicators in the training and validation cohorts.

**Nutritional-Inflammatory Indicators**	**Total N=325 (%)**	**Training cohort N=217 (%)**	**Validation cohort N=108 (%)**	***P* -value**
**Total Protein (g/L)**				0.563
< 69.80	225 (69.2%)	153 (70.5%)	72 (66.7%)	
≥ 69.80	100 (30.8%)	64 (29.5%)	36 (33.3%)	
**Albumin (g/L)**				0.179
< 43.10	225 (69.2%)	156 (71.9%)	69 (63.9%)	
≥ 43.10	100 (30.8%)	61 (28.1%)	39 (36.1%)	
**Prealbumin (mg/dL)**				0.926
< 248.00	208 (64.0%)	138 (63.6%)	70 (64.8%)	
≥ 248.00	117 (36.0%)	79 (36.4%)	38 (35.2%)	
**CREA (μmol/L)**				0.198
< 57.00	193 (59.4%)	123 (56.7%)	70 (64.8%)	
≥57.00	132 (40.6%)	94 (43.3%)	38 (35.2%)	
**RBP (mg/L)**				0.290
< 44.00	242 (74.5%)	166 (76.5%)	76 (70.4%)	
≥ 44.00	83 (25.5%)	51 (23.5%)	32 (29.6%)	
**TC (mmol/L)**				0.945
< 210.64	225 (69.2%)	151 (69.6%)	74 (68.5%)	
≥ 210.64	100 (30.8%)	66 (30.4%)	34 (31.5%)	
**BMI (kg/m²)**				0.133
Healthy-weight	239 (73.5%)	167 (77.0%)	72 (66.7%)	
Under-weight	66 (20.3%)	39 (18.0%)	27 (25.0%)	
Obesity	20 (6.15%)	11 (5.07%)	9 (8.33%)	
**CONUT scores**				0.133
≥ 2	178 (54.8%)	112 (51.6%)	66 (61.1%)	
0 - 1	147 (45.2%)	105 (48.4%)	42 (38.9%)	
**PNI**				0.340
< 51.22	238 (73.2%)	163 (75.1%)	75 (69.4%)	
≥ 51.22	87 (26.8%)	54 (24.9%)	33 (30.6%)	
**PLR**				
< 157.35	129 (39.7%)	89 (41.0%)	40 (37.0%)	0.569
≥157.35	196 (60.3%)	128 (59.0%)	68 (63.0%)	
**NLR**				0.751
< 1.67	68 (20.9%)	47 (21.7%)	21 (19.4%)	

Nutritional-Inflammatory Indicators	Total N=325 (%)	Training cohort N=217 (%)	Validation cohort N=108 (%)	*P* -value
≥ 1.67	257 (79.1%)	170 (78.3%)	87 (80.6%)	
**MLR**				0.463
< 3.58	160 (49.2%)	110 (50.7%)	50 (46.3%)	
≥ 3.58	165 (50.8%)	107 (49.3%)	58 (53.7%)	
**SII**				0.537
< 385.80	79 (24.3%)	50 (23.0%)	29 (26.9%)	
≥ 385.80	246 (75.7%)	167 (77.0%)	79 (73.1%)	
**SIRI**				0.840
< 0.36	27 (8.31%)	19 (8.76%)	8 (7.41%)	
≥ 0.36	298 (91.7%)	198 (91.2%)	100 (92.6%)	
**WBC (*10^9/L)**				0.723
< 5.90	223 (68.6%)	147 (67.7%)	32 (29.6%)	
≥ 5.90	102 (31.4%)	70 (32.3%)	76 (70.4%)	
**RBC (*10^12/L)**				0.378
< 3.67	254 (78.2%)	51 (23.5%)	20 (18.5%)	
≥ 3.67	71 (21.8%)	166 (76.5%)	88 (81.5%)	
**HGB (g/L)**				0.614
< 110	92 (28.3%)	59 (27.2%)	33 (30.6%)	
≥ 110	233 (71.7%)	158 (72.8%)	75 (69.4%)	
**PLT (*10^9/L)**				0.294
< 202	118 (36.3%)	74 (34.1%)	44 (40.7%)	
≥ 202	207 (63.7%)	143 (65.9%)	64 (59.3%)	
**Lymphocyte (*10^9/L)**				1.000
< 1.03	60 (18.5%)	40 (18.4%)	20 (18.5%)	
≥ 1.03	265 (81.5%)	177 (81.6%)	88 (81.5%)	
**Monocyte (*10^9/L)**				0.544
< 0.49	257 (79.1%)	169 (77.9%)	88 (81.5%)	
≥ 0.49	68 (20.9%)	48 (22.1%)	20 (18.5%)	
**Neutrophil (*10^9/L)**				0.665
< 2.14	40 (12.3%)	25 (11.5%)	15 (13.9%)	
≥ 2.14	285 (87.7%)	192 (88.5%)	93 (86.1%)	
**Basophil (*10^9/L)**				0.189
< 0.03	230 (70.8%)	148 (68.2%)	82 (75.9%)	
≥ 0.03	95 (29.2%)	69 (31.8%)	26 (24.1%)	
**Eosinophil (*10^9/L)**				0.704
< 0.02	303 (93.2%)	201 (92.6%)	102 (94.4%)	
≥ 0.02	22 (6.77%)	16 (7.37%)	6 (5.56%)	
**Lymphocyte (%)**				0.655
< 16.3	37 (11.4%)	23 (10.6%)	14 (13.0%)	

Nutritional-Inflammatory Indicators	Total N=325 (%)	Training cohort N=217 (%)	Validation cohort N=108 (%)	*P* -value
≥ 16.3	288 (88.6%)	194 (89.4%)	94 (87.0%)	
**Monocyte (%)**				1.000
< 5.5	64 (19.7%)	43 (19.8%)	21 (19.4%)	
≥ 5.4	261 (80.3%)	174 (80.2%)	87 (80.6%)	
**Neutrophil (%)**				0.255
< 56.8	91 (28.0%)	60 (27.6%)	31 (28.7%)	
≥ 56.8	234 (72.0%)	157 (72.4%)	77 (71.3%)	
**Basophil (%)**				0.294
< 0.40	118 (36.3%)	74 (34.1%)	44 (40.7%)	
≥ 0.40	207 (63.7%)	143 (65.9%)	64 (59.3%)	
**Eosinophil (%)**				1.000
< 4.80	284 (87.4%)	190 (87.6%)	94 (87.0%)	
≥ 4.80	41 (12.6%)	27 (12.4%)	14 (13.0%)	
**MCV (fl)**				0.100
< 91.5	207 (63.7%)	131 (60.4%)	76 (70.4%)	
≥ 91.5	118 (36.3%)	86 (39.6%)	32 (29.6%)	
**MCH (pg)**				0.335
< 30	191 (58.8%)	123 (56.7%)	68 (63.0%)	
≥ 30	134 (41.2%)	94 (43.3%)	40 (37.0%)	
**MPV (%)**				1.000
< 10.70	196 (60.3%)	65 (60.2%)	131 (60.4%)	
≥ 10.70	129 (39.7%)	43 (39.8%)	86 (39.6%)	
**PDW (%)**				0.942
< 12.30	192 (59.1%)	129 (59.4%)	63 (58.3%)	
≥ 12.30	133 (40.9%)	88 (40.6%)	45 (41.7%)	
**RDW (%)**				0.372
< 14.10	211 (64.9%)	145 (66.8%)	66 (61.1%)	
≥ 14.10	114 (35.1%)	72 (33.2%)	42 (38.9%)	
**PCT (%)**				0.379
< 0.22	123 (37.8%)	78 (35.9%)	45 (41.7%)	
≥ 0.22	202 (62.2%)	139 (64.1%)	63 (58.3%)	

Abbreviations: %, percentage; CREA, serum creatinine; RBP, retinol binding protein; TC, total cholesterol; MBI, body mass index; CONUT, controlling nutritional status; PNI, prognostic nutritional index; PLR, platelet-to-lymphocyte; NLR, neutrophil-to-lymphocyte ratio; MLR, monocyte -to-lymphocyte; SII, systemic immune inflammation index; SIRI, system inflammation response index; WBC, white blood cell count; RBC, red blood cell count; HGB, hemoglobin concentration; PLT, platelet count; MCV, mean corpuscular volume; MCH, mean corpuscular hemoglobin; MPV, mean platelet volume; PDW, platelet distribution width; RDW, red cell distribution width; PCT, platelet hematocrit;

Table S3. The definitions and calculation formulas of each nutritional-inflammatory indicator utilized in the present study.

Nutritional-Inflammatory Indicators	Formula (or Measure Unit)	Expression Level	
**Total Protein**	g/L	High (≥ 69.80)	Low (< 69.80)
**Albumin**	g/L	High (≥ 43.10)	Low (< 43.10)
**Prealbumin**	mg/dL	High (≥ 248.00)	Low (< 248.00)
**CREA**	μmol/L	High ()≥ 57.00	Low (< 57.00)
**RBP**	mg/L	High ()≥ 44.00	Low (< 44.00)
**TC**	mmol/L	High (≥ 210.64)	Low (< 210.64)
**PNI**	10 x serum albumin(g/dl) + 5 x total lymphocyte count(*10^9/L)	High (≥ 51.22)	Low (< 51.22)
**PLR**	Platelet count (*10^9/L) / Lymphocyte count(*10^9/L)	High (≥ 157.35)	Low (< 157.35)
**NLR**	Neutrophil count (*10^9/L) / Lymphocyte count(*10^9/L)	High (≥ 1.67)	Low (< 1.67)
**MLR**	Monocyte count (*10^9/L) / Lymphocyte count(*10^9/L)	High (≥ 3.58)	Low (< 3.58)
**SII**	Platelet count (*10^9/L) x Neutrophil count (*10^9/L) / Lymphocyte count(*10^9/L)	High (≥ 385.80)	Low (< 385.80)
**SIRI**	Neutrophil count (*10^9/L) x Neutrophil count (*10^9/L) / Lymphocyte count(*10^9/L)	High (≥ 0.36)	Low (< 0.36)
**WBC count**	*10^9/L	High (≥ 5.90)	Low (< 5.90)
**RBC count**	*10^12/L	High (≥ 3.67)	Low (< 3.67)
**HGB**	g/L	High (≥ 110)	Low (< 110)
**Platelet count**	*10^9/L	High (≥ 202)	Low (< 202)
**Lymphocyte count**	*10^9/L	High (≥ 1.03)	Low (< 1.03)
**Monocyte count**	*10^9/L	High (≥ 0.49)	Low (< 0.49)
**Neutrophil count**	*10^9/L	High (≥ 2.14)	Low (< 2.14)
**Basophil count**	*10^9/L	High (≥ 0.03)	Low (< 0.03)
**Eosinophil count**	*10^9/L	High (≥ 0.02)	Low (< 0.02)
**Lymphocyte**	Percentage (%)	High (≥ 16.3)	Low (< 16.3)
**Monocyte**	Percentage (%)	High (≥ 5.4)	Low (< 5.5)
**Neutrophil**	Percentage (%)	High (≥ 56.8)	Low (< 56.8)
**Basophil**	Percentage (%)	High (≥ 0.40)	Low (< 0.40)
**Eosinophil**	Percentage (%)	High (≥ 4.80)	Low (< 4.80)
**MCV**	fl	High (≥ 91.5)	Low (< 91.5)
**MCH**	pg	High (≥ 30)	Low (< 30.0)
**MPV**	Percentage (%)	High (≥ 10.70)	Low (< 10.70)
**PDW**	Percentage (%)	High (≥ 12.30)	Low (< 12.30)
**RDW**	Percentage (%)	High (≥ 14.10)	Low (< 14.10)
**PCT**	Percentage (%)	High (≥ 0.22)	Low (< 0.22)

**BMI**	kg/m²	Under-weight (< 18.5); Healthy-weight (18.5-23.9); Obesity (> 23.9)
**COUNT points**	**Albumin**, g/L (score): ≥35.0 (0); 30.0-34.0 (2); 25.0-29.0 (4); <2.5 (6)<br>**TC**, mg/dL (score) : ≥180 (0); 140-199 (1); 100-139 (4); <100 (6)<br>**Lymphocyte count**, *10^9/L (score): ≥1.6 (0); 1.20-1.59 (1); 0.80-1.19 (2); <0.8 (3)	Normal:0-1;　Mild malnutrition：2-4；<br>Moderate malnutrition：5-8；<br>Severe malnutrition：9-12；

Abbreviations: %, percentage; CREA, serum creatinine; RBP, retinol binding protein; TC, total cholesterol; MBI, body mass index; CONUT, controlling nutritional status; PNI, prognostic nutritional index; PLR, platelet-to-lymphocyte; NLR, neutrophil-to-lymphocyte ratio; MLR, monocyte -to- lymphocyte; SII, systemic immune inflammation index; SIRI, system inflammation response index; WBC, white blood cell count; RBC, red blood cell count; HGB, hemoglobin concentration; PLT, platelet count; MCV, mean corpuscular volume; MCH, mean corpuscular hemoglobin; MPV, mean platelet volume; PDW, platelet distribution width; RDW, red cell distribution width; PCT, platelet hematocrit;

Table S4. Results of variance inflation factor (VIF) of regression analysis.

Final variables	GVIF	Df	GVIF^(1/(2*Df))
**Basophil Count**	1.13	1	1.06
**PLR**	1.06	1	1.03
**CA125**	1.10	1	1.04
**SCC-Ag**	1.06	1	1.03
**CONUT scores**	1.23	1	1.11
**Histological Type**	1.03	1	1.02
**FIGO Stage**	1.07	2	1.02

Abbreviations: PLR, platelet-to-lymphocyte; carcinoembryonic antigen; CA125, cancer antigen 125; SCC-Ag, squamous cell carcinoma antigen; CONUT, controlling nutritional status; FIGO, International Federation of Gynecology and Oncology.

Table S5. NRI and IDI of the nomogram in survival prediction compared with established Models.

.

Index	NRI		IDI	
	**Estimate (95% CI)**	***P*-value**	**Estimate (95% CI)**	***P*-value**
**Vs. Model1**				
For 5-year OS	0.34 (0.14-0.53)	0.012	1.00 (0.03-0.20)	0.004
**Vs. Model2**				
For 5-year OS	0.35 (0.06-0.49)	0.016	0.11 (0.03-0.21)	0.008
**Vs. Model3**				
For 5-year OS	0.21 (0.03-0.47)	0.024	0.10 (0.03-0.20)	<0.001
**Vs. Model4**				
For 5-year OS	0.32 (0.09-0.52)	<0.001	0.11 (0.04-0.23)	<0.001
**Vs. Model5**				
For 5-year OS	0.15 (0.07-0.27)	0.004	0.40 (0.21-0.57)	<0.001

Prognostic indicators of age, histological type, FIGO staging system and PLR were introduced into model1[24]. Prognostic indicators of age, histological type, FIGO staging system and MLR were introduced into model2[24]. Prognostic indicators of age, histological type, FIGO staging system and NLR were introduced into model3[24]. Prognostic indicators of age, histological type, FIGO staging system and PNI were introduced into model4[24]. Prognostic indicators of FIGO staging system, platelet count and neutrophils count were introduced into model5[32].
